# Nanoformulation-Based 1,2,3-Triazole Sulfonamides for Anti-*Toxoplasma In Vitro* Study

**DOI:** 10.3390/tropicalmed8080401

**Published:** 2023-08-07

**Authors:** Fadwa M. Arafa, Heba Said, Doaa Osman, Nadjet Rezki, Mohamed R. Aouad, Mohamed Hagar, Mervat Osman, Bassma H. Elwakil, Mariusz Jaremko, Mona Mohamed Tolba

**Affiliations:** 1 Department of Medical Parasitology, Faculty of Medicine, Alexandria University, Alexandria 21577, Egypt; 2 Department of Parasitology, Medical Research Institute, Alexandria University, Alexandria 21561, Egypt; 3 Department of Chemistry, College of Science, Taibah University, Al Madinah Al Munawarah 30002, Saudi Arabia; 4 Department of Chemistry, Faculty of Science, Alexandria University, Alexandria 21321, Egypt; 5 Department of Medical Laboratory Technology, Faculty of Applied Health Sciences Technology, Pharos University in Alexandria, Alexandria 21526, Egypt; 6 Smart-Health Initiative (SHI) and Red Sea Research Center (RSRC), Division of Biological and Environmental Sciences and Engineering (BESE), King Abdullah University of Science and Technology (KAUST), Thuwal 23955, Saudi Arabia

**Keywords:** chitosan nanoparticles, *Toxoplasma gondii*, 1,2,3-triazole, sulfonamides, in vitro studies

## Abstract

*Toxoplasma gondii* is deemed a successful parasite worldwide with a wide range of hosts. Currently, a combination of pyrimethamine and sulfadiazine serves as the first-line treatment; however, these drugs have serious adverse effects. Therefore, it is imperative to focus on new therapies that produce the desired effect with the lowest possible dose. The designation and synthesis of sulfonamide-1,2,3-triazole hybrids (**3a–c**) were performed to create hybrid frameworks. The newly synthesized compounds were loaded on chitosan nanoparticles (CNPs) to form nanoformulations (**3a.CNP**, **3b.CNP**, **3c.CNP**) for further in vitro investigation as an anti-*Toxoplasma* treatment. The current study demonstrated that all examined compounds were active against *T. gondii* in vitro relative to the control drug, sulfadiazine. **3c.CNP** showed the best impact against *T. gondii* with the lowest IC_50_ value of 3.64 µg/mL. Using light microscopy, it was found that Vero cells treated with the three nanoformulae showed remarkable morphological improvement, and tachyzoites were rarely seen in the treated cells. Moreover, scanning and transmission electron microscopic studies confirmed the efficacy of the prepared nanoformulae on the parasites. All of them caused parasite ultrastructural damage and altered morphology, suggesting a cytopathic effect and hence confirming their promising anti-*Toxoplasma* activity.

## 1. Introduction

The protozoan parasite *Toxoplasma gondii* is an Apicomplexan with a life cycle including both sexual and asexual reproduction. Cats are the only definitive hosts for *T. gondii,* nevertheless*,* it infects a wide spectrum of intermediate hosts, ranging from birds and rodents to humans, making it one of the world’s most efficient parasites [1]. Toxoplasmosis is now the second leading cause of mortality from food-borne illness [2]. *T. gondii* has expanded globally, and it is estimated that 30% of humans have been infected with *T. gondii* [3].

*T. gondii* infection is divided into two phases: acute infection and chronic infection. *T. gondii* multiplies quickly in hosts as tachyzoites, which can be trapped by the host’s immune response during the acute infection stage. Then, the infection progresses to the chronic phase, during which tachyzoites transform to bradyzoites, a slowly reproducing stage of the parasite. This stage leads to the generation of tissue cysts in order to evade the immune response, thereby establishing persistent infection in the muscles, heart, or central nervous system (CNS) [4]. Humans can become infected with *T. gondii* through a variety of means, including contaminated food or water, vertical transmission, or organ transplants. Toxoplasmosis in immune competent hosts is typically asymptomatic and treatment is rarely required. However, it causes serious illness in congenitally infected children, resulting in severe consequences such as blindness and diverse neurological ailments in developing fetuses and newborns as well as in immunologically suppressed humans, particularly recipients of organ transplant and AIDS patients [5].

Currently, the most efficient therapy for toxoplasmosis is a combination of pyrimethamine and sulfadiazine, which work synergistically to block folic acid production. However, this combination has considerable adverse effects, including hypersensitivity, bone marrow suppression, and teratogenic consequences [6,7]. Spiramycin is employed to treat congenital toxoplasmosis alone or in combination with sulfadiazine and pyrimethamine. The combination of these medications has undesirable side effects, such as bone marrow suppression, which can be alleviated by supplementation with folic acid [8]. There is currently no pharmacological therapy for toxoplasmosis that provides desirable benefits, such as a medication that can cross biological barriers, target a single objective, and reduce harmful side effects [9]. As a consequence, toxoplasmosis therapy must now focus on achieving the most effective target with the lowest feasible dose, thereby minimizing side effects. This can be accomplished by utilizing an appropriate delivery mechanism. Although sulfadiazine has relatively good tissue permeability, its poor water solubility may limit its absorption [10,11].

Molecular hybridization is a simple way to discover new drugs in which two peculiar active pharmacophores are linked together [12,13,14]. This approach is most commonly used in developing new pharmacological scaffolds that target multiple sites. Consequently, the resulting hybrid molecules can dramatically reduce the risk of drug interactions and multiple drug resistance. We reveal our interest in the synthesis of such hybrid compounds and the examination of their synergistic impact in order to retain interest in this research and because the tunable 1,2,3-triazole core and sulfonamide moieties have enormous biological significance [15,16,17,18,19,20,21,22].

Nanoparticles, on the other hand, attracted more attention after demonstrating their ability to improve drug pharmacokinetic profiles, which includes increasing solubility, dissolving rate, stability, and, most importantly, changing drug permeability through absorption into membranes, resulting in lower medication dosages [23]. One of the main goals of using nanoformulations is to perform better drug penetration through biological barriers such as the blood-brain barrier (BBB) [24]. Over the last several decades, chitosan has received a great deal of attention and has been extensively used in drug research in light of its good biological qualities, such as biocompatibility, absorptivity, non-hypersensitivity, biodegradability, and wound-healing capabilities [25]. Previous studies have revealed that chitosan nanoparticles have anti-protozoal properties such as anti-*Giardia* [26], and anti-*Toxoplasma* properties [27]. The unique attributes of chitosan nanoparticles might indeed exceed their affinity for negatively charged biological membranes and specific site targeting in vivo [28]. They have been demonstrated to have non-toxic effects on normal human liver cells while having dose-dependent inhibitory effects on the proliferation of several tumor cell lines [29].

To prepare anti-*Toxoplasma* medications that are incredibly successful and have few dangerous side effects, several research organizations have participated in a wide range of innovative chemical development against *T. gondii*. As a result of these discoveries, we expect to be able to customize 1,2,3-triazole-sulfonamide molecular conjugates and their binding on chitosan nanoparticles in a way that can almost exactly imitate their anti-protozoal capability. Focused 1,2,3-triazole-sulfonamide hybrids were synthesized again [30] and loaded onto chitosan nanoparticles to create newer formulations in a nonmetric size in order to increase the drug’s surface area, biological activity, bioavailability, and permeability. These formulations were then rationalized and tested for their anti-*Toxoplasma* potency.

## 2. Materials and Method

### 2.1. Chemistry

All solvents and **reagents** used were of the highest analytical reagent grade and were not purified further. The melting points were determined using the Stuart Scientific SMP1 and uncorrected. TLC was performed on UV fluorescent Silica gel Merck 60 F254 plates, with spots identified using a UV lamp (254 nm). The main functional groups were identified using a SHIMADZU FTIR~Affinity~1S spectrometer with a range of 400~4000 cm^−1^. NMR spectra were collected using a Bruker spectrometer (400 MHz) with tetramethyl silane (TMS) as an internal reference. The LCMS/MS impact II was used for high-resolution mass spectroscopy (HRMS). Elemental analyses were carried out using a Vario EL III Elementar Analyzer. The nanoparticles used in this investigation were prepared according to our previous study [31].

### 2.2. General Method for the Synthesis Sulfonamide-Based 1,2,3-Triazoles 3a–c

A solution of copper sulfate (0.10 g) and sodium ascorbate (0.15 g) in water (10 mL) was added dropwise to a solution of propargyl amine (1 mmol) in DMSO (10 mL). Then, suitable sulfa drug azide **2a–c** (1 mmol) was added to the reaction mixture under stirring, which was continued for 6–10 h at room temperature. After completion of the reaction (as monitored by thin layer chromatography (TLC) in hexane-ethyl acetate), crushed ice water was added to the mixture. The resulting precipitate was collected by filtration, washed with saturated ammonium chloride solution, and then recrystallized from ethanol/DMF to provide the necessary 1,2,3-triazoles **3a–c**.

***Characterization of 4-(4-(aminomethyl)-1H-1,2,3-triazol-1-yl)-N-(pyrimidin-2-yl)benzenesulfonamide (3a)***. It was obtained as yellow powder in 86% yield. Mp: 220~221 °C. IR (KBr: ύ_max_): 1560 (C=C), 1620 (C=N), 2940 (CH.Al), 3045 (CH.Ar), 3380~3450 cm^−1^ (NH, NH_2_). ^1^H~NMR (400 MHz, DMSO~*d*_6_): δ_H_ = 12.02 (1H, s, N**H**SO_2_), 8.50 (1H, s, **H**~5~triazolyl), 8.16~8.43 (6H, m, Ar~**H**), 7.04 (1H, bs, Ar~**H**), 6.84 (2H, s, N**H_2_**), 4.25 (2H, s, C**H_2_**). ^13^C~NMR (100 MHz, DMSO~*d*_6_): δ_C_ = 159.25, 158.64, 150.23, 146.36, 139.25, 128.33, 127.85, 122.75, 121.44, 120.12, 119.97 (Ar~**C, C**=N); 56.56 (**C**H_2_). HRMS (ESI): 331.0654 [M^+^]. Calculated for C_13_H_13_N_7_O_2_S: C: 47.12; H; 3.95; N; 29.59. Found: C: 47.29; H, 3.78; N, 29.34.

***Characterization of 4-(4-(aminomethyl)-1H-1,2,3-triazol-1-yl)-N-(pyridine-2-yl)benzenesulfonamide (3b)***. It was obtained as yellow solid in 84% yield. Mp: 248~250 °C. IR (KBr: ύ_max_): 1550 (C=C), 1610 (C=N), 2920 (CH_al_), 3060 (CH_ar_), 3350~3440 cm^−1^ (NH, NH_2_). ^1^H~NMR (400 MHz, DMSO~*d*_6_): δ_H_ = 12.43 (1H, s, N**H**SO_2_), 8.88 (1H, s, **H**~5~triazolyl), 8.39 (2H, bs, Ar~**H**), 7.76~8.05 (4H, m, Ph~**H**), 7.23~7.51 (2H, m, Ar~**H**), 6.85 (1H, s, N**H_2_**), 4.42 (2H, s, NC**H_2_**). ^13^C~NMR (100 MHz, DMSO~*d*_6_): δ_C_ = 159.75, 158.07, 154.11, 148.76, 140.45, 129.45, 128.22, 123.87, 122.11, 121.22, 119.73 (Ar~**C, C**=N); 56.49 (**C**H_2_). HRMS (ESI): 330.0689 [M^+^]. Calculated for C_14_H_14_N_6_O_2_S: C: 50.90; H: 4.27; N: 25.44. Found: C: 50.78; H: 4.42; N: 25.67.

***Characterization of N-(diaminomethylene)-4-(4-(aminomethyl)-1H-1,2,3-triazol-1-yl)benzenesulfonamide (3c)***. It was obtained as yellow pale powder in 88% yield. Mp: 278~279 °C. IR (KBr: ύ_max_): 1580 (C=C), 1615 (C=N), 2960 (CH_al_), 3080 (CH_ar_), 3310~3460 cm^−1^ (NH_2_). ^1^H~NMR (400 MHz, DMSO~*d*_6_): δ_H_ = 8.72 (s, 1H, **H**~5~triazolyl), 7.94~8.07 (m, 4H, Ar~**H**), 6.78 (bs, 6H, 3 x N**H_2_**), 4.43 (s, 2H, NC**H_2_**). ^13^C~NMR (100 MHz, DMSO~*d*_6_): δ_C_ = 158.45, 157.29, 155.45, 156.26, 147.45, 138.39, 128.23, 126.66, 124.87, 121.22, 121.09, 118.87, (Ar~**C**, **C**=N); 57.09 (**C**H_2_). HRMS (ESI): 295.0599 [M^+^]. Calculated for C_10_H_13_N_7_O_2_S: C: 40.67; H: 4.44; N: 33.20. Found: C: 40.49; H: 4.67; N: 33.35.

### 2.3. Preparation of Nanoformulated Sulfa Drugs

A suspension of chitosan (0.5 g) (100–150 kDa, DDa ≈ 85%, Sigma-Aldrich, Saint Louis, MO, USA) in acetic acid solution (100 mL; 2% *v*/*v*) was stirred for 30 min and then filtered using Whitman filter paper no. 1. After mixing sodium tripolyphosphate (TPP) (0.2% *w*/*v*) with a solution of triazole-sulfonamide derivative **3a~c** (40 mg) in DMSO (10 mL), the resulting mixture was added dropwise to the chitosan solution with continuous stirring for 30 min, and then centrifuged (25,000 rpm for 20 min). The resulting precipitate was kept for further analysis at 4 °C in sterile falcon tubes.

### 2.4. Characterization of Nanoformulae

The particle size (PS), polydispersity index (PDI), and ζ potential of the prepared nanoformulae were measured using the dynamic light scattering technique with a Malvern Zetasizer. Meanwhile, the ultrastructure, size, and shape of the prepared nanoformulae were detected by transmission electron microscopy (TEM) [32]. The resulting nanoformulae were lyophilized and weighed before further analyses.

### 2.5. Determination of Loading and Entrapment Efficiencies

The loading and entrapment efficiencies of the produced nanoformulae were evaluated by diluting each nanoformulation with phosphate-buffered solution (PBS) at a ratio of 1 to 10 *v*/*v*, followed by centrifugation of the diluted samples for 15 min at 15,000 rpm. The proportion of loaded and unentrapped drug in each supernatant was determined independently using UV/Vis spectroscopy (Spekol 1300, Analytik Jena, Jena, Germany), with methanol serving as a blank (at 280 nm) [32]. The percentage of the entrapment efficiency (*EE*%) was calculated according to Equation (1), while the percentage of the loading efficiency (*LE*%) was calculated according to Equation (2):

*EE*% = Total initial drug − Total unentrapped drug/Total initial drug × 100 (1)

*LE*% = Total entrapped drug/Nanoparticle weight × 100 (2)

### 2.6. Maintenance of Toxoplasma Strain

The virulent *T. gondii* RH strain utilized in this investigation was maintained in the Medical Parasitology Department at the Faculty of Medicine at Alexandria University via multiple intraperitoneal passages into Swiss albino mice. On the fifth day after inoculation, peritoneal exudates were extracted, the tachyzoites were passed through a 27-gauge needle, cleaned twice, and centrifuged for 10 min at 1000× *g* in RPMI 1640 (Gibco BRL, Karnataka, India) without fetal bovine serum (FBS). Lastly, the parasites were suspended in the same medium at 10^6^ parasites per milliliter. Viability was assessed using 0.2% Trypan blue stain [33,34].

### 2.7. Vero Cell Line

Vero cells (kidney fibroblast cells of African green monkeys) were acquired from the National Cancer Institute in Cairo, Egypt, and were kept at the tissue culture section of the Medical Research Institute in Alexandria, Egypt. Cell line culture was carried out using RPMI 1640 supplemented with 10% FBS (Gibco BRL) and 1% penicillin-streptomycin solution (Gibco BRL). The cells were incubated at 37 °C with 5% CO_2_ humidity [35].

### 2.8. Cytotoxicity Tests

The cytotoxicity of the prepared compounds **3a–c**, their nanoformulae (**3a–c.CNP**)**,** the chitosan nanoparticles (**CNP**), and sulfadiazine (positive control) on Vero cells was investigated using the 3-(4,5-dimethylthiazol-2-yl)-2,5-diphenyl tetrazolium bromide (MTT) assay. The compounds were initially prepared in DMSO as stock solutions and then diluted 100 times with culture media to obtain the first concentration. Vero cells were cultured at a density of 10^4^ cells per well in a 96-well plate. The plate was incubated at 37 °C in a humidified 5% CO_2_ incubator. After 24 h, cells were treated with serial dilutions of each compound, where the prepared compounds **3a–c**, their nanoformulae **3a–c.CNP, CNP,** and sulfadiazine were dissolved in RPMI-1640 culture medium. Three replicates for each drug concentration were performed and cell viability was assessed using the MTT assay. Twenty microliters of 5 mg/mL MTT solutions (Sigma, Saint Louis, MO, USA) was added to each well and the plate was incubated at 37 °C for 3 h. Then, the MTT solution was removed, 100 µL DMSO was added, and the optical density was measured at 570 nm using a Benchmark Microplate Reader (Bio Rad, **Karnataka, India**). Cytotoxicity, expressed as CC_50_, was defined as the concentration of test sample that caused 50% destruction of cells [36]. Vero cell viability (%) was estimated using the following equation:

$$\mathrm{Viability}\%=\frac{\mathrm{The}\;\mathrm{absorbance}\;\mathrm{of}\;\mathrm{cells}\;\mathrm{treated}\;\mathrm{with}\;\mathrm{nano}\;\mathrm{sulfadrug}}{\mathrm{The}\;\mathrm{absorbance}\;\mathrm{of}\;\mathrm{cells}\;\mathrm{cultured}\;\mathrm{with}\;\mathrm{medium}\;\mathrm{alone}}\times 100$$

Finally, **CC_50_** was calculated using CompuSyn software (version 1, ComboSyn Inc., Paramus, NJ, USA) [37,38].

### 2.9. In Vitro Growth Inhibition Assay

Vero cells were cultured in 96-well plates (10^4^ cells/well) in RPMI 1640 medium supplemented with 10% inactivated FBS at 37 °C with 5% CO_2_. After 24 h, the cells were infected with *T. gondii* tachyzoites (parasite: cell ratio = 10:1). After 4 h period of incubation, the extracellular parasites were removed by triple washing with culture media. 24 h later, the cells were treated with serial dilutions of each compound (the prepared compounds **3a–c**, their nanoformulae **3a–c.CNP, CNP**, and sulfadiazine were dissolved in DMSO stock solutions and individual dilutions with culture media (100×) were performed to obtain the first concentration). The MTT assay, as mentioned before, was applied to assess the viability of cells. Growth inhibition (GI) was calculated using the following equation:

GI (%) = [(At − Ac)/Ac] × 100

where At and Ac are the absorbances of the treated cells and control, respectively. The half-maximal inhibitory concentration (IC_50_) values were obtained using CompuSyn software (version, 1) [37,38]. The selectivity index (SI) of each tested nanoformulation was calculated using the resultant IC_50_ from the in vitro growth inhibition assay and the **CC_50_** value from the cytotoxicity profile, where SI = CC_50_/IC_50_ [36].

### 2.10. The Effect of Nanoformulae on Non-Infected and Infected Vero Cell Line under Light Microscopy

The Vero cell line was cultivated on the glass coverslips of 10 dishes (35 mm cell culture dish) until confluent. The cells on five coverslips were infected with 1 × 10^4^ tachyzoites of *T. gondii* and incubated for 4 h, then the glass coverslips were washed with phosphate- buffered saline solution (Gibco BRL). Following 24 h incubation, the three prepared nano-formulations**,** sulfadiazine (positive control), and RPMI media (negative control) were added to five dishes in a dose of 50 µg/mL for 24 h at 37 °C. Finally, the glass coverslips were washed twice and fixed with methanol prior to staining with Giemsa (Sigma-Aldrich Corp, **Saint Louis, MO, USA**). Another five dishes were incubated with the three nanoformulae, sulfadiazine, and RPMI media for 24 h. After incubation, the glass coverslips were washed twice, fixed with methanol, and stained with Giemsa (Sigma-Aldrich Corp). All the prepared samples were observed under a light microscope. Qualitative analysis methods were used to observe the effect of the tested nanoformulae on both non-infected and infected cell cultures in comparison to positive and negative controls [39].

### 2.11. Scanning Electron Microscopy (SEM)

The ultrastructure of *T. gondii* tachyzoites treated in vitro with nanoformulae **3a–c.CNP** was detected using SEM (Joel JSM-53001A, Tokyo, Japan) to further explore their anti*-Toxoplasma* effects. Tachyzoites from peritoneal exudates of infected mice on the fifth day post-inoculation were obtained as previously described [34]. Tachyzoites were separated into four tubes, each containing 10^5^ cells. The first tube acted as the control (normal, untreated group), while the remaining three tubes each received individual doses of nanoformulae **3a–c.CNP**. After that, the tachyzoites were incubated for two hours at room temperature. Later, the tachyzoites were rinsed in cacodylate buffer, fixed with 2% paraformaldehyde and 2.5% glutaraldehyde in 0.1 M sodium cacodylate buffer (pH 7.4), and mounted on a slide. The slide was dehydrated using graded ethanol dilutions (70%, 80%, 90%, and 100%) and post-fixed for 2–4 h using 1–2% osmium tetroxide in 0.1 M phosphate buffer (pH 7.2). The slides were subsequently placed on stubs, dried using the critical point procedure, coated with gold (20–30 nm), and observed by SEM [40].

### 2.12. Transmission Electron Microscopy (TEM)

Following Vero cell confluence in four T-25 culture flasks, *T. gondii* RH strain tachyzoites were suspended in 5 mL of RPMI and added to each flask at a parasite–host cell ratio of 5:1 [41]. The flasks were incubated for 2 h. The cells were then washed twice with culture medium to eliminate extracellular parasites. The cells in culture flasks were incubated in 5 mL of culture medium for 24 h at 37 °C in a 5% CO_2_ environment [42]. The first flask was left untreated, while the other three flasks were treated for 24 h with the IC_50_ of each nanoformulation (6.14, 4.93, and 3.64 µg/mL for **3a.CNP**, **3b.CNP**, and **3c.CNP**, respectively). Trypsinization was followed by centrifugation at 2000 × *g* for 10 min, and the resultant pellet was fixed 2.5% glutaraldehyde in phosphate buffer and kept at 4 °C until utilized [43]. The fixed specimens were carefully cleaned with Millonig’s phosphate buffer before being post-fixed with osmium tetroxide-phosphate buffer. They were then dehydrated at increasing concentrations of ethyl alcohol before being embedded in epoxy resin. Finally, ultrathin slices were stained twice with uranyl acetate and lead citrate trihydrate stains before being inspected under TEM (Jeol JSM-1400, **JEOL, Tokyo, Japan**) [44].

## 3. Results

### 3.1. Chemistry

**Scheme 1:**
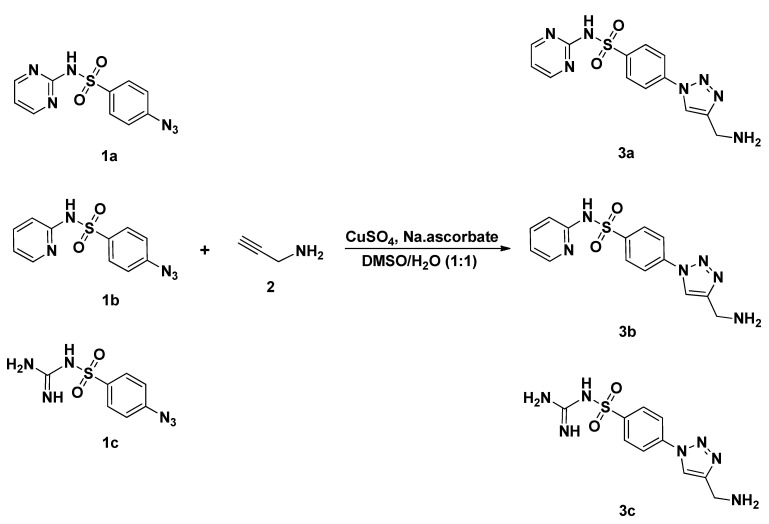
Click synthesis of sulfonamide-1,2,3-triazole molecular conjugates **3a–c**.

Scheme 1 shows the effective synthesis of 1,2,3-triazole-based sulfonamides using the Cu(I)-click chemistry strategy [45]. Two complementary building blocks that include an azide side chain and a terminal alkyne were needed to perform the “click” 1,3-dipolar cycloaddition reaction. The sulfa medicines **1a–c** (widely available) first went through well-known diazotization and azidolysis processes, yielding only one of the important matching intermediates (sulfonamide azide derivatives **2a–c**) [46]. The copper (I) catalyzed 1,3-dipolar cycloaddition reaction of the freshly synthesized azides **2a–c** and propargyl amine yielded regioselectivity of the targeted 1,2,3-triazole-sulfonamide molecular hybrids **3a–c**. In the presence of the catalytic amount of copper sulfate and sodium ascorbate and a mixture of DMSO/water as solvent, the click reactions were carried out at room temperature (Scheme 1).

The structures of the ensuing 1,2,3-triazole-sulfonamide molecular conjugates **3a–c** were inferred from the spectrum data. The absence of ≡C-H and C≡C in their IR spectra demonstrated that they were involved in the cycloaddition process. The spectra also showed that there were new characteristic absorption bands at 3310–3460 cm^−1^ that were attributed to the amino groups (NH, NH_2_).

The ^1^H NMR spectra of click adducts **3a–c** clearly indicated the disappearance of the signal attributed to the precursor acetylenic proton (≡C-H) of the respected propargyl amine and the appearance of a distinct singlet at δ_H_ 8.50–8.88 ppm, which was assigned to the **H**-5 triazolyl proton. In addition, the spectra also revealed the presence of characteristic singlets at δ_H_ 4.25–4.42, 6.78–6.84, and 12.02–12.43 ppm related to NC**H_2_**, N**H_2_**_,_ and N**H**SO_2_ protons, respectively. The aromatic protons were also recorded in their respected aromatic region (see experimental section).

Furthermore, the success of the dipolar cycloaddition process was further supported by the ^13^C NMR spectra. The disappearance of the signals attributable to the two sp carbons (**C**≡**C**) was plainly seen in every spectrum. Additionally, the NCH_2_ carbons were responsible for the signals recorded at ppm values of 56.56 to 57.09, while aromatic carbons were responsible for the signals recorded at ppm values of 118.87 to 159.75.

### 3.2. Characterization of Nanoformulae

**Figure 1:**
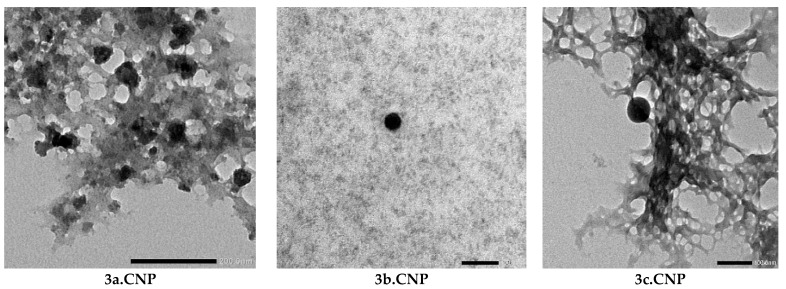
Transmission electron microscope study of **3a–c.CNP**.

The newly synthesized sulfa drug-loaded chitosan nanoparticles (**3a–c.CNP**) were prepared using the reported ionic gelation technique [31]. The data shown in Table 1 demonstrated that the loaded nanoparticles had a positive charge, indicating the stability of the created nanoformulae that may have been related to completion of loading the analogues. The produced nanoparticles had a homogeneous, smooth, spherical form and the particles sizes were in the range of 87.5, 43.2, and 56.8 nm for **3a.CNP**, **3b.CNP**, and **3c.CNP**, respectively, as demonstrated in Figure 1. The best entrapment and loading efficiencies were observed with **3c.CNP** nanoparticles (83.4% and 29.4%, respectively) (Table 1).

### 3.3. Cytotoxicity Tests

Regarding the effect of the treated Vero cells with different concentrations of the prepared compounds **3(a–c)**, their nanoformulae **3a–c.CNP**, **CNP**, and sulfadiazine, the percentage of viable cells was used to calculate (CC_50_). The results showed that **CC_50_** values of the three nanoformulations, **3a.CNP, 3b.CNP,** and **3c.CNP**, were equal to 157.64, 123.17, and 145 µg/mL, respectively, while those of the prepared compounds, 3a, 3b, and 3c, were equal to 218, 168, and 155 µg/mL respectively. The chitosan nanoparticles showed the highest **CC_50_** value, approximately 1597 µg/mL, indicating that they were less toxic than the referenced sulfadiazine used in this study, which had a **CC_50_** value of 348.55 µg/mL, as shown in Table 2.

### 3.4. In Vitro Growth Inhibition Assay

**Figure 2:**
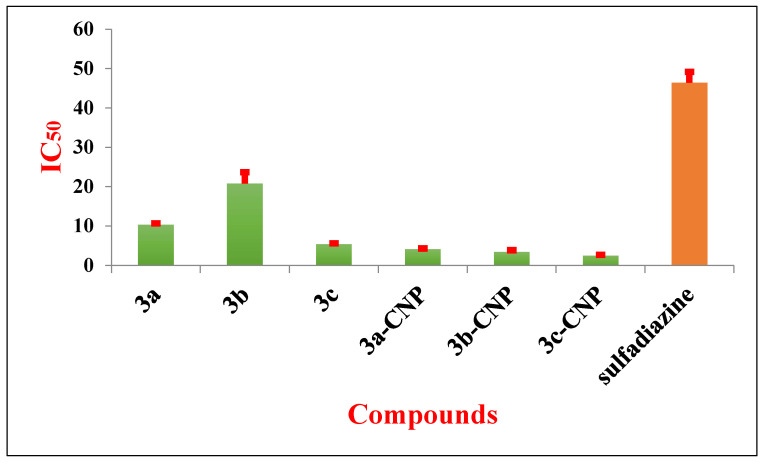
The IC_50_ concentrations (μg/mL) of **3a–c**, their nanoformulae (**3a–c.CNP**), and sulfadiazine against *T. gondii* tachyzoites.

The anti-*Toxoplasma* ability of the prepared compounds **3a–c**, their nanoformulae **3a–c.CNP**, the chitosan nanoparticles **CNP**, and sulfadiazine was investigated. Within 24 h after treatment, the effectiveness of the studied compounds to prevent intracellular tachyzoite growth in Vero cells was evaluated using the MTT assay. The results were tabulated and are shown in Table 2 and Figure 2, respectively. Each prepared compound **3a–c**, their nanoformulae **3a–c.CNP,** and the chitosan nanoparticles **CNP** had more potent activity against *T. gondii* at lower concentrations with respect to that displayed by the sulfadiazine drug control. The synthesized nanoformulae (**3a–c.CNP**) were found to be more effective against *T. gondii* than the prepared compounds **3a–c**, with IC_50_ values of 6.14, 4.93, and 3.64 µg/mL for nanoformulae **3a–c.CNP** respectively, while the IC_50_ values of **3a–c** were 10.35, 20.78, and 5.39 µg/mL, respectively. The chitosan nanoparticles alone had moderate activity against *T. gondii*, with an IC_50_ value 14.2 µg/mL, while an IC_50_ value of 46.42 µg/mL was recorded for sulfadiazine, the control drug.

In regard to the results of the present investigation of the SI* of the studied compounds, the nanoformulae showed higher anti-*T. gondii* activity than their corresponding synthesized sulfa drugs **3a–c** and also higher than sulfadiazine (the positive control drug), as illustrated in Table 2. Consequently, they were selected for additional investigations to illustrate their effects and the mechanism of action by light microscopy, SEM, and TEM.

### 3.5. The Effect of Nanoformulae on Non-Infected and Infected Vero Cell Line under Light Microscopy

**Figure 3:**
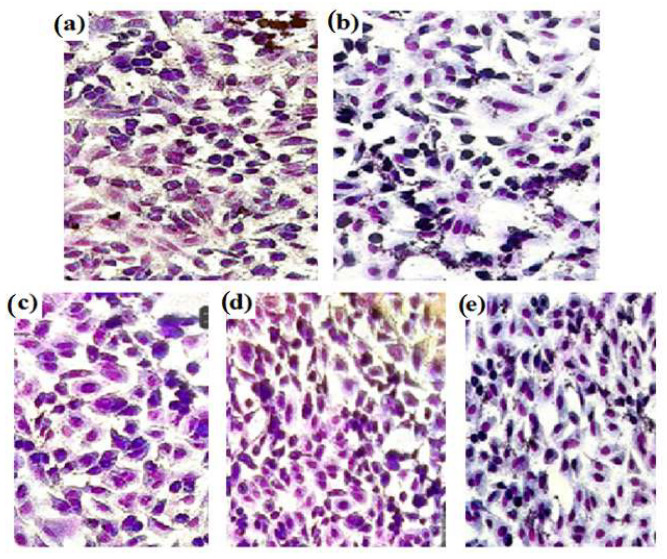
Morphology of Vero cells after 24 h of exposure to 50 μg/mL of (**a**) **RPMI medium**, (**b**) **sulfadiazine**, (**c**) **3a.CNP**, (**d**) **3b.CNP** and (**e**) **3c.CNP**. Stained using Giemsa stain (×200).

The effects of the three new nanoformulae **3a–3c.CNP** on non-infected Vero cells under light microscopy are shown in Figure 3. This figure highlights the fact that, when exposed to 50 μg/mL of **3a–3c.CNP** and sulfadiazine for 24 h, Vero cells did not display a significant difference in morphology or confluence compared to the control (Figure 3a–e).

**Figure 4:**
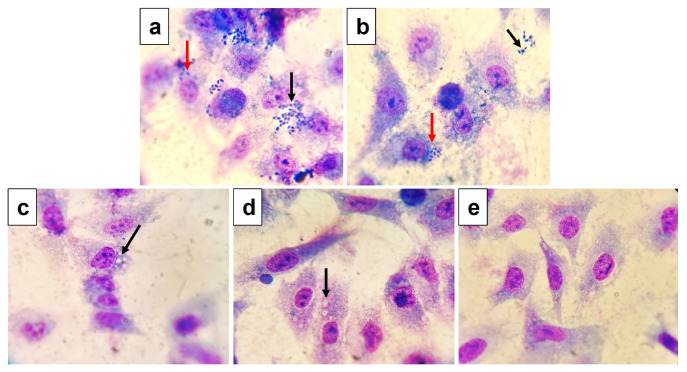
Morphology of Vero cells infected with *T. gondii* after 24 h of exposure to (**a**) **RPMI medium:** the tachyzoites of *T. gondii* can be seen inside the inoculated Vero cells (red arrow), as well as adhered to the glass coverslip (black arrow); and 50 μg/mL each of (**b**) **sulfadiazine:** the tachyzoites can be seen inside (red arrow) and outside (black arrow) treated Vero cells, (**c**) **3a.CNP**, (**d**) **3b.CNP**, and (**e**) **3c.CNP**: *T. gondii* tachyzoites were rarely seen in the infected cells; however, some vacuoles, representing degenerated parasites, were noticed (black arrows). Stained using Giemsa stain (×1000).

As predicted, the light microscopy results illustrated that infected, non-treated Vero cells showed host cell lysis, regression, reattachment, and invasion by parasites, with many tachyzoites noticed inside and outside the cells, as shown in Figure 4a. On the other hand, infected cells treated with 50 μg/mL of sulfadiazine contained parasites with similar morphology to untreated cells, as illustrated in Figure 4b. Vero cells subjected to the three nanoformulae showed higher confluence than cells treated with sulfadiazine (the positive control) and continued to grow with remarkable improvement in their morphology, as expected. Furthermore, after treatment with 50 μg/mL of each nanoformulation**,** the number of tachyzoites decreased dramatically, and *T. gondii* tachyzoites were rarely seen in the infected cells; however, some vacuoles, representing degenerated parasites, were noticed (Figure 4c–e).

### 3.6. Scanning Electron Microscopy (SEM)

To deeply understand the anti-*Toxoplasma* mode of action of the prepared nanoformulae of sulfa drugs, the structure of the tachyzoites treated with nanoformulae **3a–3c.CNP** was compared with that of normal, non-treated tachyzoites by SEM.

**Figure 5:**
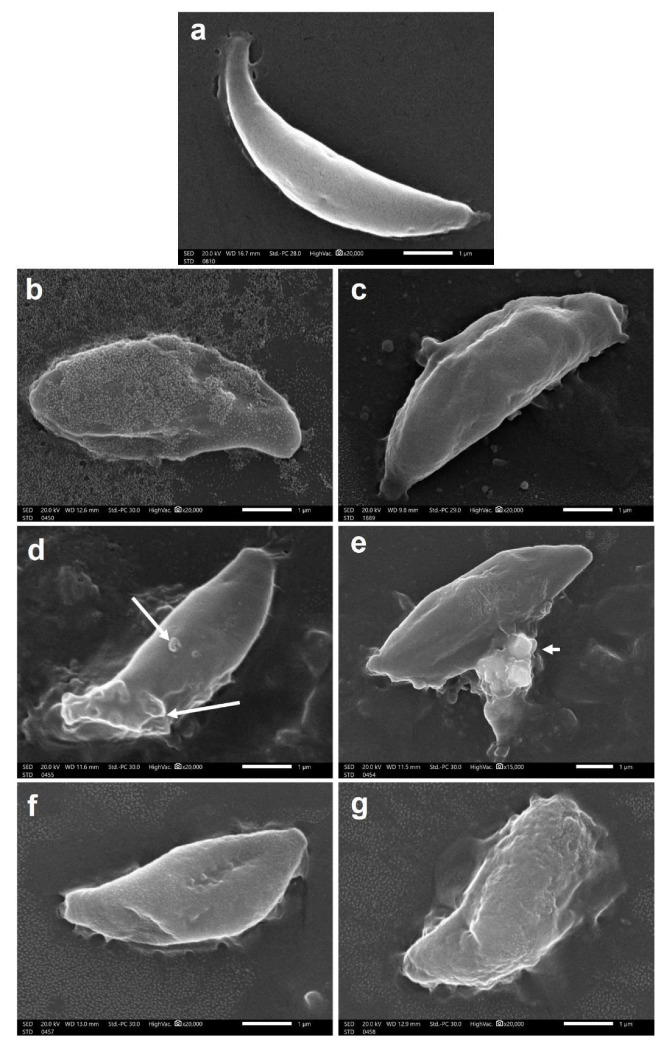
SEM of *T. gondii* tachyzoites (**a**) Regular, non-treated tachyzoite with tapered anterior end and rounded posterior end (×20,000). (**b**,**c**) Tachyzoites treated with **3a.CNP** showing rough outer surface with multiple depressions and deep furrows (×20,000). (**d**,**e**) Tachyzoites treated with **3b.CNP** showing multiple surface blebs (arrows) and loss of surface integrity with leakage of cytoplasmic contents (arrowhead) (×20,000). (**f**,**g**) Tachyzoites treated with **3c.CNP** showing multiple surface depressions and completely damaged and disfigured morphology (×20,000).

The non-treated tachyzoites exhibited crescent-shaped parasites with a tapered anterior end and normal rounded posterior end (Figure 5a). Tachyzoites exposed to **3a.CNP** showed a rough outer surface with multiple depressions and deep furrows (Figure 5b,c), whereas those treated with **3b.CNP** lost their smooth surface, with the appearance of multiple surface blebs, loss of surface integrity, and leakage of cytoplasmic contents (Figure 5d,e). Finally, tachyzoites treated with **3c.CNP** showed multiple surface depressions and a completely damaged and disfigured morphology (Figure 5f,g).

### 3.7. Transmission Electron Microscopy (TEM)

**Figure 6:**
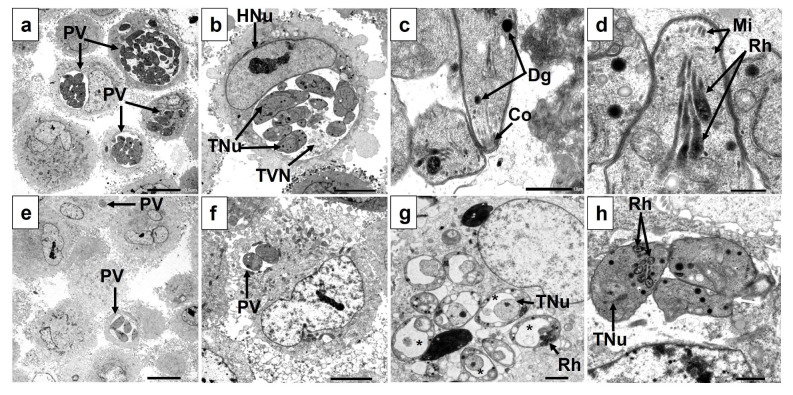
Transmission electron microscopic (TEM) images of *Toxoplasma gondii*-infected, non-treated (**a**–**d**) and **3a.CNP**-treated (**e**–**h**) Vero cells showing: (**a**) Multiple infected, non-treated cells showing several parasitophorous vacuoles (PV) containing a large number of tachyzoites (×600). (**b**) A cross-section of an infected cell where the host cell’s nucleus (HNu) and a large PV containing multiple tachyzoites can be seen. Each tachyzoite contained a clearly delineated nucleus (TNu) and multiple organelles and was surrounded by the structures of the tubulovesicular network (TVN) (×1500). (**c**,**d**) A longitudinal section of the anterior end of the tachyzoites showing conoid (Co), dense granules (Dg), micronemes (Mi), and thin elongated rhoptry bulbs (Rh) (×8000 and ×12,000, respectively). (**e**,**f**) Infected, **3a.CNP**-treated cells showed fewer infected cells and PVs with fewer tachyzoites inside them (×600 and ×1500, respectively). (**g**) A cross-section of an infected, **3a.CNP**-treated cell containing multiple vacuolated tachyzoites where the nuclei (TNu) can be seen surrounded by vacuoles (asterisks) containing degraded materials. The rhoptries (Rh) can be seen to be pushed aside by the vacuoles. Destabilized PV membrane with infiltration of the host cell’s cytoplasmic contents within the PV (×2000). (**h**) Tachyzoite treated with **3a.CNP** showing swollen rhoptry bulbs (Rh) and the nucleus (TNu) lost its lining nuclear membrane. Other tachyzoites appeared tethered to each other (white arrow) with no clear line of demarcation between them (×5000).

Transmission electron microscopic (TEM) examination was executed after a 24 h period of treatment to ascertain the underlying mechanism of action of the three sulfa drugs loaded chitosan nanoparticles (**3a–c.CNP**) on the intracellular tachyzoites. *Toxoplasma gondii*-infected, non-treated cells had intact nuclei and multiple infected cells were detected in the field with several parasitophorous vacuoles (PVs) containing a large number of tachyzoites (Figure 6a). The normal tubulovesicular network surrounding the tachyzoites could also be detected in the intravacuolar space. Each tachyzoite had an intact plasma membrane, a clearly delineated nucleus, and multiple organelles (Figure 6b). The apical complex structures could be detected, including dense granules, micronemes, and rhoptries with thin elongated bulbs (Figure 6c,d).

On the hand, there were fewer infected cells after treatment with all nanoformulae as demonstrated with **3a.CNP**, and the PVs had fewer tachyzoites inside them compared to infected, non-treated cells (Figure 6e,f). The tachyzoites were hugely vacuolated with vacuoles containing degraded materials surrounding the nuclei and pushing the rhoptries aside. Destabilized PV membranes with infiltration of the host cell’s cytoplasmic contents within the PV could be detected. The structures of the tubulovesicular network could not be clearly visualized (Figure 6g). Some tachyzoites showed swollen rhoptry bulbs and the nuclei lost their delineating nuclear membranes. Meanwhile, other tachyzoites appeared tethered to each other with no clear line of demarcation between them (Figure 6h).

**Figure 7:**
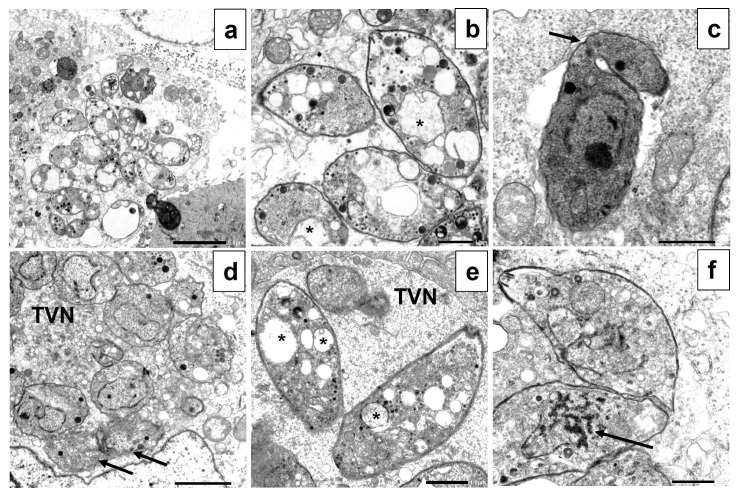
Transmission electron microscopic (TEM) images of *Toxoplasma gondii*-infected, **3b.CNP**-treated (**a**–**c**) and **3c.CNP**-treated (**d**–**f**) Vero cells showing: (**a**) Multiple, **3b.CNP**-treated tachyzoites appeared extremely vacuolated without nuclei or organelles (×1500). (**b**) At higher magnification, tachyzoites appeared to have lost their nuclei and all apical complex structures. They had multiple-sized vacuoles (asterisks) containing degraded materials (×5000). (**c**) Tachyzoites treated with **3b.CNP** were tethered to each other (black arrow) (×8000). (**d**) Multiple intracellular, **3c.CNP**-treated tachyzoites within a PV. Some appeared apparently normal, while others had hazy cytoplasmic membranes (black arrows) without nuclei or organelles. The tubulovesicular network (TVN) appeared dark and granular with an absence of the PV membrane (×4000). (**e**) Tachyzoites treated with **3c.CNP** appeared without any nuclei or organelles, and only multiple-sized vacuoles (asterisk) containing materials at different stages of degradation were seen. The tubulovesicular network (TVN) appeared dark and granular (×6000). (**f**) Tachyzoites treated with **3c.CNP** were severely destructed without nuclei or organelles. Nuclear chromatin could be detected in the cytoplasm without any delineating nuclear membrane (black arrow). A triangular structure resembling the anterior end of another superimposed degenerated tachyzoite could be seen (white arrow) (×6000).

Meanwhile, **3b.CNP**-treated tachyzoites appeared extremely vacuolated without nuclei or organelles (Figure 7a). At higher magnification, the tachyzoites appeared to have lost their nuclei and all apical complex structures. They had multiple-sized vacuoles containing degraded materials (Figure 7b). Some tachyzoites were tethered with each other by their ends (Figure 7c). Finally, following **3c.CNP** treatment, some of the intracellular tachyzoites had hazy cytoplasmic membranes without nuclei or organelles. The tubulovesicular network appeared dark and granular with an absence of the PV membrane (Figure 7d). Other tachyzoites were severely destructed without any nuclei or organelles, and only multiple-sized vacuoles containing degraded materials were seen (Figure 7e). Nuclear chromatin could be detected in the cytoplasm without any delineating nuclear membrane. Additionally, a triangular structure resembling the anterior end of another superimposed degenerated tachyzoite could be seen (Figure 7f).

## 4. Discussion

*T. gondii* is one of the most common and widespread parasites in the world and has a wide range of warm-blooded hosts, such as birds, rodents, and humans. Even without any symptoms, this parasite infects nearly half of the human population [47]. Several experts find this parasite’s treatment to be challenging since this tachyzoites can penetrate the blood-brain barrier (BBB), producing deadly encephalitis [48]. A pharmacological treatment for toxoplasmosis that achieves the desired results might include a molecule that can penetrate biological barriers, address a particular target, and reduce harmful side effects. Hence, the search for innovative therapeutic approaches has to be carefully monitored in hopes of creating an effective and well-tolerated treatment [49]. Many studies have been conducted using NPs as drug or vaccine vehicles to improve their therapeutic efficacy [31].

The discovery of anti-parasitic drugs requires the investigation of molecules with the ability to kill parasites but not their host. Scientific evidence documenting the anti-parasitic properties of compounds is initially derived from in vitro studies. The capacity to audit the anti-parasitic activity of these substances is the major benefit of in vitro studies. It is crucial to make sure the substances are safe for the host and will not have any negative effects on the host’s functionality [50,51].

Molecular hybridization could offer a promising opportunity to develop novel compounds with encouraging anti-parasitic activity. Significant anti-parasitic impact might be achieved by including the triazole moiety of the sulfadiazine medication alongside amino groups [14]. Dzitko et al. [52] investigated the effect of triazole-based compounds on *Toxoplasma* in mouse fibroblast culture, and the results revealed that triazole-based compounds were four times more effective than sulfadiazine while also being less toxic to the host. Furthermore, it was discovered that triazole compounds most likely target purine nucleoside phosphorylase (PNPase) [52]. The generation of de novo purine nucleotides and ribose phosphate strands hinges on this enzyme [53,54]. Triazole-based compounds were discovered to inhibit the PNPase enzyme, thus reducing purine bases and causing parasite death [54].

To ensure proper drug delivery at minimum concentration, 1,2,3-triazole-sulfonamide hybrids (**3a–c**), developed in the current study, were loaded into CNP. The resultant NP characterization revealed homogenous nanoparticles with increased size upon drug loading from 40 nm to a maximum of 87.5 nm when the triazole-sulfa drug hybrids were loaded on chitosan NPs. This smaller particle size could justify their metabolic activity, in which the sizes of the prepared formulae may enhance tissue bypass [55]. Additionally, the PDI values were <0.5, which indicated the homogeneity of the prepared form (monodispersed) [56].

Moreover, the Zetasizer results showed that the formulated nanoparticles had a positive ζ potential charge due to the hydrogen bonds between the amino group and hydroxyl groups of chitosan [57]. The positive charge was seen as an added value of the produced NPs because it would enhance the ability of the CNPs to readily move across the negative channels of the cell membrane [58]. Enhanced entrapment efficiency increased with both reduced particle size and larger positive potential. Additionally, it was noted that less aggregation occurred inside the infected cells due to the high positive potential of the nanoparticles. The high positive ζ potential of the prepared CNPs (+30 to +36 mV) was also an illustration of their stability since stability was found to improve at greater positive potentials, i.e., >30 mV [59]. Finally, the higher the positive ζ potential in accordance with the lower vesicle size improved the entrapment efficiency.

In regard to the current study, the nanoformulae of sulfa drugs were tested for potential cytotoxic activity against Vero cells in vitro. The results showed that the CC_50_ values of the three nanoformulae **3a.CNP**, **3b.CNP**, and **3c.CNP** were equal to 157.64, 120.17, and 141 µg/mL, respectively. The cells were viable at concentrations up to 100 μg/mL and had no remarkable changes, which is an indication of their safety for the cell line at much higher values than the required IC_50_.

Following 24 h of treatment, the MTT assay was used to assess the capacity of the tested nanoformulae (**3a.CNP**, **3b.CNP**, and **3c.CNP**) to limit intracellular tachyzoite growth in Vero cells. The absorbance determines the number of surviving Vero cells. Since parasites harm living Vero cells during invasion and proliferation, the absorbance indirectly evaluates the parasite-inhibiting effects of medications. All three of the nanoformulae—**3a.CNP**, **3b.CNP**, **3c.CNP**—and CNP had more anti-*T. gondii* activity than sulfadiazine, which acted as a positive pharmacological control. The compound **3c.CNP** was demonstrated to be the most effective against *T. gondii*, with an IC_50_ value of 3.64 g/mL, while sulfadiazine was found to be the least effective, with an IC_50_ value of 49.794 g/mL.

A widely utilized metric to express a compound’s anti-*Toxoplasma* activity is the selectivity index (SI*), and only substances with higher SI* values can be deemed to be more selective candidates [60]. Theoretically, the higher SI* ratio, the greater the efficiency against *T. gondii*. The nanoformulation **3c.CNP** had the highest SI* value among all tested compounds. This may be due to the small particle size and highest entrapment and loading efficiencies.

Microscopic techniques were used to observe cell morphology and enhance the understanding of the in vitro anti-parasitic activity. Light microscopy clearly showed that the addition of each nanoformulation to the Vero cell monolayers had no impact and the cells remained metabolically active and viable. On the other hand**,** it was also used in the present study to recognize the clampdown of *T. gondii* growth by sulfadiazine and the investigated nanoformulae **3a–c.CNP**. Tachyzoites could be seen both inside and outside the infected, non-treated Vero cells. The substantial changes in Vero cell morphology and confluence observed in the group treated with RPMI medium only (negative control) were most likely due to rapidly proliferating *T. gondii* tachyzoites propagating through invasion of Vero cells, thus causing host cell lysis, regression, and reattachment [61]. It also indicated that the tested nanoformulae efficiently inhibited the growth of *T. gondii* better than the control drug, sulfadiazine, as the number of tachyzoites decreased dramatically after adding the drugs. Additionally, *T. gondii* tachyzoites were rarely seen in the infected cells, although some vacuoles, representing degenerated parasites, were noticed after treatment. In addition, the nanoformulae were more effective than sulfadiazine against cell proliferation and confluence was greater. These findings confirmed that the nanoformulae had higher anti-*Toxoplasma* activity and also lacked any sort of selective toxicity against the infected host cell [31].

Further, SEM was used to properly assess the morphological changes induced by the investigated 1,2,3-triazole-based nanoformulae (**3a–c.CNP**) in the treatment of tachyzoites. It was observed that non-treated tachyzoites appeared to be crescent-shaped with a pointed anterior end and a blunt posterior end; additionally, the surface was smooth, regular, and complete. It was clear that most tachyzoites treated with the nanoformulae had deep dimples, furrows, and ridges on the surfaces; in addition, some tachyzoites demonstrated leakage of their cytoplasmic contents. The tachyzoite ultrastructural alterations were more illustrated in tachyzoites treated with **3c.CNP**, where the cells appeared completely disrupted. Transmission electron microscopic (TEM) examination augmented the data provided by SEM. Moreover, it helped theorize the most probable mechanisms of action of the three assessed nanoformulae on the intracellular tachyzoites over a period of 24 h of treatment.

Notably, all three of the tested nanoformulae resulted in a significant ultrastructural change in the form of cytoplasmic vacuoles, which occurred hand in hand with disruption of the parasite nuclei and organelles. These vacuoles contained materials at different stages of degradation. The vacuoles resembled those previously reported by Souto et al. as structures suggestive of autophagy (SSA) [62]. The tested nanoformulae caused several changes to apical complex organelles involved in tachyzoite invasion into target cells, ranging from disrupting them to their complete disappearance. The invasion process is an important virulence factor of *T. gondii* tachyzoites, being an obligatory intracellular parasite; therefore, targeting apical complex organelles can prevent the invasion process and thus prevent parasite dissemination [63]. Intracellular multiplication is another function that helps the parasite increase its numbers to spread into neighboring cells. The tethered tachyzoites might suggest that the effect in already established infection was due to deformed daughter cell budding, leading to blocked intracellular development and eventually parasite dissemination [64,65]

The most drastic changes were noticed with compound **3c.CNP**, where tachyzoites were completely disrupted with complete disappearance of organelles and lysis of the nuclear membrane. The tubulovesicular network appeared dark and granular due to the release of cytoplasmic contents out of the disrupted cytoplasmic membranes of the affected tachyzoites [64], this together with a destabilized PV membrane might have allowed permeation of the host cell’s cytoplasmic contents into the PV [62].

Previous research reported that treatment with sulfadiazine produces no significant morphological changes to the parasite; however, treatment causes a decline in the number of intracellular parasites, which is known as a cytostatic effect [66,67]. In contrast, the tested nanoformulae in the current study caused parasite growth reduction as well as damaged and altered morphology, suggesting a cytopathic effect. Some of the noticed ultrastructural changes resembled those reported by previous studies. Giovati et al. reported some of the apoptotic changes in treated tachyzoites, which included cytoplasmic vacuolation and disruption of the nuclear membrane [68]. Moreover, other studies explored the autophagy process and reported that the presence of autophagic vacuoles in the cytoplasm, the disappearance of cytoplasmic structures and PV membranes, progressive degeneration of the parasites, and nuclear disintegration in *T. gondii* tachyzoites were hallmarks of autophagy [69,70]. The simultaneous existence of different types of cell death has been described in other parasites, such as *Trypanosoma cruzi* [71], *Leishmania infantum* [72], and *T. gondii* [73]. Therefore, autophagy and apoptosis could be both possible mechanisms of death after treatment with nanoformulae **3a–c.CNP**.

## 5. Conclusions

Three focused chitosan-loaded 1,2,3-triazole-sulfonamide nanoparticles **3a–c.CNP** were successfully synthesized, characterized, and investigated for their anti*-T. gondii* potency. The results showed that **3a–c.CNP** all exhibited great anti-*Toxoplasma* action while still being safe for regular Vero cells, but the **3c.CNP** derivative was found to be the most potent agent due to its **IC_50_** value, which was 15 times lower than the **IC_50_** of sulfadiazine. All of the nanoformulae caused parasite growth reduction as well as ultrastructural damage and altered morphology, suggesting a cytopathic effect. Eventually, these findings revealed promising anti-*Toxoplasma* activity and could be interesting to investigate in vivo in order to further understand the mechanism of action as a promising treatment for toxoplasmosis.

## Figures and Tables

**Table 1:** Physicochemical characterization of **3a–c.CNP,** particle size (**PS**), zeta potential (ζ potential), percentage of the entrapment efficiency (***EE*%**), percentage of loading efficiency (***LE*%**), and polydispersity index (***PDI***).

Nano Formulae	PS (nm)	ζ Potential (mV)	*PDI*	Entrapment Efficiency % (*EE*%)	Loading Efficiency (*LE*%)
**3a.CNP**	87.5 ± 5.4	+30.3 ± 1.0	0.23 ± 0.07	82.1 ± 0.8	27.23 ± 1.7
**3b.CNP**	43.2 ± 8.1	+36.4 ± 0.1	0.33 ± 0.13	81.3 ± 0.3	22.9 ± 1.0
**3c.CNP**	56.8 ± 9.2	+34.4 ± 0.3	0.41 ± 0.05	83.4 ± 1.1	29.4 ± 1.0

**Table 2:** In vitro cytotoxicity, anti-*Toxoplasma* activity, selectivity index of the prepared materials **3(a–c)**, their nanoformulae **3a–c.CNP**, and the chitosan nanoparticles (**CNP)** vs. sulfadiazine.

Tested Drug	CC_50_ (µg/mL)	IC_50_ (µg/mL)	SI
**3a**	218.8 ± 1.104	10.35 ± 0.27	21
**3b**	168.0 ± 9.300	20.78 ± 2.85	8.11
**3c**	155.0 ± 3.355	5.39 ± 0.20	28.75
**3a.CNP**	157.64 ± 1.332	6.14 ± 0.263	25.67
**3b.CNP**	123.17 ± 2.99	4.93 ± 0.662	24.37
**3c.CNP**	145.0 ± 5.900	3.64 ± 0.295	38.736
**CNP**	>1000	14.2 ± 1.75	ND
**sulfadiazine**	361.45 ± 15.531	46.42 ± 2.77	7.79

## Data Availability

The data presented during the current study are available from the corresponding author on request.
